# Development of the Breast Surgical Oncology Fellowship in the United States

**DOI:** 10.1155/2022/3342910

**Published:** 2022-05-19

**Authors:** Lauren Zammerilla Westcott, Ronald C. Jones, James W. Fleshman

**Affiliations:** Department of Surgery, Baylor University Medical Center, Dallas, Texas, USA

## Abstract

The surgical treatment of breast cancer has rapidly evolved over the past 50 years, progressing from Halsted's radical mastectomy to a public campaign of surgical options, aesthetic reconstruction, and patient empowerment. Sparked by the research of Dr. Bernard Fisher and the first National Surgical Adjuvant Breast and Bowel Project trial in 1971, the field of breast surgery underwent significant growth over the next several decades, enabling general surgeons to limit their practices to the breast. High surgical volumes eventually led to the development of the first formal breast surgical oncology fellowship in a large community-based hospital at Baylor University Medical Center in 1982. The establishment of the American Society of Breast Surgeons, as well as several landmark clinical trials and public campaign efforts, further contributed to the advancement of breast surgery. In 2003, the Society of Surgical Oncology (SSO), in partnership with the American Society of Breast Surgeons and the American Society of Breast Disease, approved its first fellowship training program in breast surgical oncology. Since that time, the number of American fellowship programs has increased to approximately 60 programs, focusing not only on training in breast surgery, but also in medical oncology, radiation oncology, pathology, breast imaging, and plastic and reconstructive surgery. This article focuses on the happenings in the United States that led to the transition of breast surgery from a subset of general surgery to its own specialized field.

## 1. Introduction: The Breast Surgery Movement

Breast cancer awareness has become a social and cultural movement. Individuals worldwide understand the meaning of the “pink ribbon” as a symbol of support for breast cancer and its patients, survivors, and research. This international recognition is appropriate, as breast cancer is the world's most prevalent cancer. At the end of 2020, there were 7.8 million women alive who were diagnosed with breast cancer in the last five years. [1] The treatment of breast cancer has radically changed over the past several decades, leading to the development of breast surgery as a surgical specialty. As only 50 years have passed since the first National Surgical Adjuvant Breast and Bowel Project (NSABP) clinical trial in 1971, it is fruitful to analyze the contributions and historical happenings that have led to the development of breast surgery as a specialty.

## 2. 1970-1971: Dr. Bernard Fisher's Surgical Dilemma and Landmark Trial

**Figure 1:**
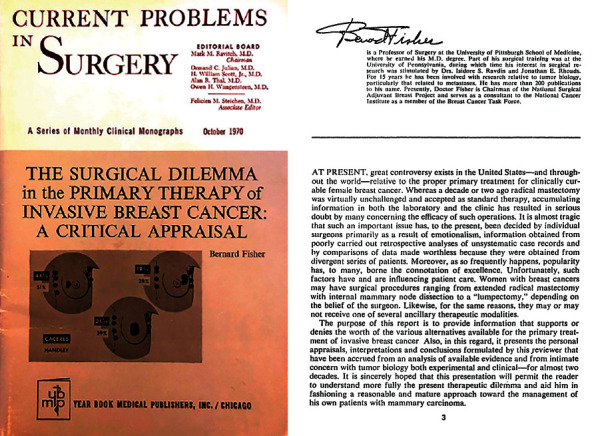
Current problems in surgery, October 1970. Dr. Bernard Fisher first publishes his “alternate hypothesis” regarding tumor metastasis, challenging the radical mastectomy.

Prior to 1970, the “Halstedian Hypothesis” governed the way that physicians thought about cancer and metastasis. This paradigm proposed an orderly spread from local to distant sites [2]. However, even after a “radical” resection of skin, breast, muscle, and lymphatics, patients succumbed to widespread breast cancer. It was at this time that Dr. Bernard Fisher from the University of Pittsburgh published a critique entitled “The Surgical Dilemma in the Primary Therapy of Invasive Breast Cancer: A Critical Appraisal” (Figure 1).

In this essay, Fisher presented the results of his laboratory studies, which demonstrated that tumor dissemination is not dictated by anatomic considerations but by a host-tumor relationship that determined its virulence and metastatic propensity. He proposed this as an “alternate hypothesis,” thus presenting the first challenge to the Halsted radical mastectomy as the standard operation for the treatment of breast cancer [3, 4]. As chairman of the NSABP in 1971, Fisher decided to test his “alternate hypotheses” in a randomized prospective clinical trial. NSABP-B04 compared the Halsted radical mastectomy with total mastectomy (preserving the pectoralis major, pectoralis minor, and axillary lymph nodes) along with total mastectomy followed by irradiation. This study confirmed that among women with negative nodes, there was no difference in disease-free survival, relapse-free survival, distant-disease-free survival, or overall survival between those who received total mastectomy or radical mastectomy [5]. The study also did not demonstrate an advantage of adding local-regional radiation to total mastectomy, supporting the paradigm shift to less radical surgery for breast cancer [6]. The results of the B-04 trial were so significant that in 1979 the National Institutes of Health Consensus Conference announced that a modified radical mastectomy and axillary dissection should be considered the standard treatment for early breast cancer, resulting in the rapid decline in radical mastectomy [7].

Dr. Bernard Fisher's findings are a milestone in cancer treatment, as he showed that less radical surgery plus chemotherapy or radiation therapy accomplished the goal with much less morbidity. In the context of cancer treatment, Fisher helped demonstrate that surgical procedures can be tailored to the availability of other treatments. While prior to Fisher's studies, surgery was the only option and a minority of patients could be cured by surgical removal of their tumors alone, his findings allowed cancer surgery to become more effective, with less morbidity [8].

## 3. 1974: The Diagnosis and Candor of First Lady Betty Ford

**Figure 2:**
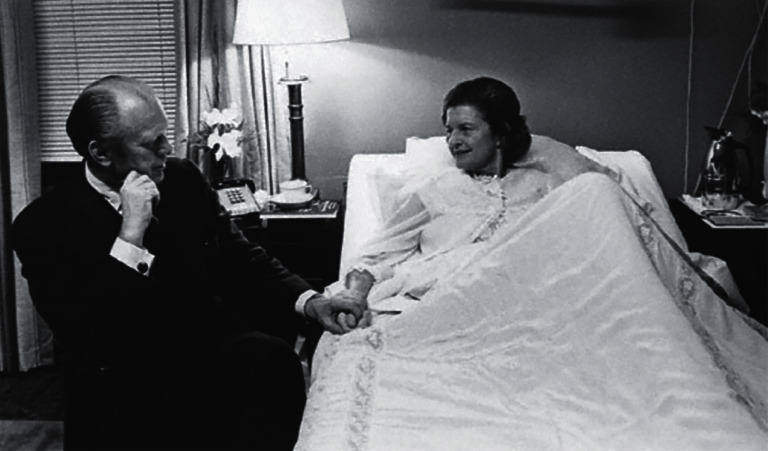
President Ford visits First Lady, Betty Ford, at Bethesda Naval Hospital after her mastectomy, 1974. Betty Ford's openness about her diagnosis of breast cancer brought the disease into the public sphere.

Even while changes were being made in the treatment of breast cancers based on clinical research, the public discussion of breast cancer was not typically promoted or accepted. Betty Ford, the First Lady in 1974, set an unusual precedent by sharing her new diagnosis of breast cancer with the country [9]. She encouraged women to “come out into the open” about the emotional and psychological toll that breast cancer could take and advocated for early detection and screening. After her diagnosis and treatment, the number of women getting mammograms increased dramatically, as did the number of women willing to talk about their own diagnosis [10]. Her openness was also a catalyst for further breast cancer research. The raised public awareness created momentum around breast cancer and women's health, providing the research community the publicity it needed to move forward (Figure 2).

## 4. 1976-1977: Breast Conservation Surgery and the Approval of Endocrine Therapy

In 1976, Fisher and the NSABP launched the B-06 trial, comparing lumpectomy and axillary lymph node dissection with or without total breast irradiation to total mastectomy and axillary lymph node dissection in patients with stages I and II breast cancers, 4 centimeters or less. At both 8 and 12 years of follow-up, lumpectomy with or without irradiation did not demonstrate a difference in disease-free survival, distant-disease-free survival, or overall survival when compared to total mastectomy [11, 12]. At 12 years of follow-up, the incidence of tumor recurrence in the ipsilateral breast was 35% in the group treated with lumpectomy alone and 10% in the group treated with lumpectomy and breast irradiation, demonstrating a statistically significant difference [12]. The B-06 trial, therefore, was critical for establishing the concept of breast-conserving therapy and confirming radiation as a component of such treatment [6]. Subsequent clinical trials, specifically B-09 and B-14, established that the use of tamoxifen in patients whose tumors contained estrogen receptors was beneficial in both node-positive and node-negative breast cancer [3]. These studies contributed to the U.S. Food and Drug Administration's approval of the drug tamoxifen for metastatic tumors in 1977. Studies showed that survival advantages occurred when a combination of chemotherapy and hormonal agents was used as adjuncts to surgical treatment. Furthermore, radiation techniques were being refined. The treatment of breast cancer was revolutionized, shifting away from the historic mutilating surgery towards breast conservation, improved survival, and improved quality of life [9]. This shift to selective therapy in breast cancer influenced the treatment paradigms of treatment for colon, rectal, anal, and esophageal cancers.

## 5. 1978–1982: Breast Surgery Specialization and the First Breast Fellowship

**Figure 3:**
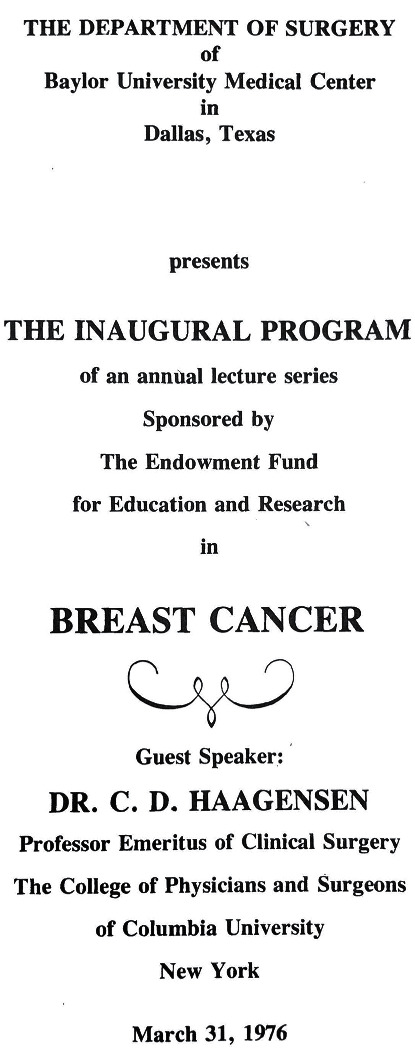
Program from the 1976 Inaugural Breast Lectureship held at Baylor University Medical Center, featuring guest speaker Dr. C. D. Haagensen. The lectureship has been held every year since its inauguration.

As the incidence and intricacies of breast cancer continued to rise, general surgeons started to realize that specialization in breast surgery was feasible. With a population just shy of one million people in the late 1970s, Dallas, Texas, had reached a size that allowed Dr. J. Harold Cheek to limit his practice to disease of the breast. Although Dr. Cheek had a general surgery practice at Baylor University Medical Center for over twenty years, he had always been interested in breast surgery, giving focused talks on breast disease and management [13]. By limiting his practice to breast surgery, he became one of the few surgeons in the country to specialize in the field. Dr. Cheek not only wanted to specialize in breast surgery but also strived to educate others about the work being done in breast diseases at the time. Thus, he helped start the first breast lectureship at Baylor in 1976 (Figure 3).

Over the next few years, the volume of breast cancer patients at Baylor significantly increased, and Dr. Cheek felt that a breast fellowship could be supported. He noted that many surgical residents were completing their training without significant experience in the surgical treatment of breast cancer [9]. Although he initially met resistance from the general surgery residency program, Dr. Cheek emphasized that he wanted to educate on the “care of the breast patient, the surgery, the pathology, and all that goes with it, not just surgery alone.” [13] On Thanksgiving Day in 1980, Dr. Cheek's vision became reality through the generous endowment of one of his prior breast cancer patients, Hannah Seeger Davis, who was the daughter of prominent surgeon Stanley Joseph Seeger [14]. This financial contribution led to the development of what was aptly named the Seeger Endowed Fellowship in Surgical Oncology of the Breast. The fellowship, which matched its first fellow in 1982, became the first fellowship in the Department of Surgery at Baylor University Medical Center and to our knowledge the first formal breast surgical oncology fellowship in a large community-based hospital.

## 6. 1984–1994: HER2, BRCA, and the Multidisciplinary Approach to Breast Cancer Treatment

The identification of the HER2 receptor in 1984 launched a new wave of breast cancer research, focusing on tumor biology. Research by Dr. Timothy Eberlein demonstrated an overexpression of the HER2 receptor in breast and ovarian cancers, providing a target receptor of interest that was recognized by cytotoxic T lymphocytes [15]. The trajectory of medical oncology, therefore, became the combination of four disciplines–genetics, immunology, pathology, and pharmacology–as a multidisciplinary approach to treatment was adopted [16]. The eventual development of Trastuzumab, a monoclonal antibody against HER2, led to the initiation of multiple clinical trials investigating the survival benefit of the drug when used in women with HER2 positive tumors [17]. The notion of hereditary breast cancer gained further scientific support with identification of the tumor suppressor genes BRCA1 and BRCA2 in 1994-1995. Their significance lay in the possibility that identification of the genes could result in closer surveillance and earlier detection for high-risk family members [16]. Additional research on BRCA revealed the link to other cancers, such as ovarian cancer, raising the awareness of the interrelationship of reproductive organs and cancer in the female patient.

The multidisciplinary approach to the treatment of breast cancer sparked the development of the first breast surgery centers. Dr. Susan Love, a female pioneer in breast surgery at the time, founded the Faulkner Breast Center–the first in the country staffed entirely by women physicians. By 1992, Dr. Love was recruited to UCLA, where she developed a model multidisciplinary breast center [18]. Another female pioneer in breast cancer, Dr. Sonya Eva Singletary, was appointed chief of the Section of Breast Cancer at M.D. Anderson in 1990, a post she held for over a decade. In 1992, she was named to serve on the President's Cancer Panel Special Commission on Breast Cancer to assess the status of breast cancer research, detection, treatment, and prevention throughout the United States [19].

## 7. 1995–2001: The Origin and Growth of the American Society of Breast Surgeons

Screening programs for breast cancer in women were in full swing in the early 1990s. This resulted in a natural shift towards the earlier diagnosis of breast cancer, resulting in an increase in identification of nonpalpable disease. Advancements in imaging techniques and procedural capabilities resulted in the development of radiologic guided biopsy and diagnosis of breast cancer. Consequently, surgeons noticed a decline in the number of surgical breast biopsies, as patients preferred a less invasive procedure for diagnosis [9].

Breast surgeons saw the need to establish standards for breast cancer diagnosis and management. Thus, in 1995, the American Society of Breast Surgeons (ASBrS) was established to promote the previously mentioned goals. The mission of the ASBrS is to encourage the study of breast surgery, promote research and development of advanced surgery techniques, improve standards of practice for breast surgery in the United States, and serve as a forum for the exchange of ideas. As a leadership organization, it also aims to advocate for surgeons who seek excellence in the care of breast patients [20]. This advocacy came to light in 1996 when the society lobbied to defeat legislation in Texas that would have prevented surgeons from performing stereotactic breast biopsies [21]. Furthermore, the ASBrS made tremendous efforts on the surgeon's behalf to resolve differences with the American College of Radiology, establishing joint credentialing for image-guided breast biopsy [21]. The society, which began with 200 members, grew to 2000 members in a matter of ten years, making it the fastest growing surgical society in the country at that time. Given such rapid growth, the ASBrS was awarded a seat on the Board of Governors of The American College of Surgeons in 2001 [21].

## 8. 2003: The Society of Surgical Oncology Approves Its First Breast Oncology Fellowship

While institutions such as Baylor University Medical Center and other major medical centers had previously established breast surgery fellowships in the prior decade, the fellowships were not formally approved by a governing organization. At the turn of the twenty-first century, the Society of Surgical Oncology (SSO), which had sponsored a surgical oncology fellowship since 1983, was seeking to expand. In 2003, in partnership with the American Society of Breast Surgeons and the American Society of Breast Disease, the SSO approved its first fellowship training program in breast oncology. The University of Texas Southwestern Medical Center became the first program to be site visited and approved [22]. The SSO upholds specific guidelines and requirements, reviewing and approving breast oncology fellowship training programs yearly. The general educational objectives include dedicated time in breast surgery, medical oncology, radiation oncology, pathology, breast imaging, and plastic and reconstructive surgery to provide an in-depth understanding of each of these disciplines [23]. The number of fellowship programs has increased to approximately 60 programs since 2003 [24].

Under the SSO, the Breast Surgical Oncology Fellowship is a separate entity from the Complex General Surgical Oncology Fellowship. The fellowships have separate match processes, and there is variation among institutions whether they support one or both SSO fellowships. Thus, the overlap between breast and general surgical oncology fellows is institution specific. For example, while Baylor University Medical Center supports a breast fellowship, the institution does not have a general surgical oncology fellowship.

**Figure 4:**
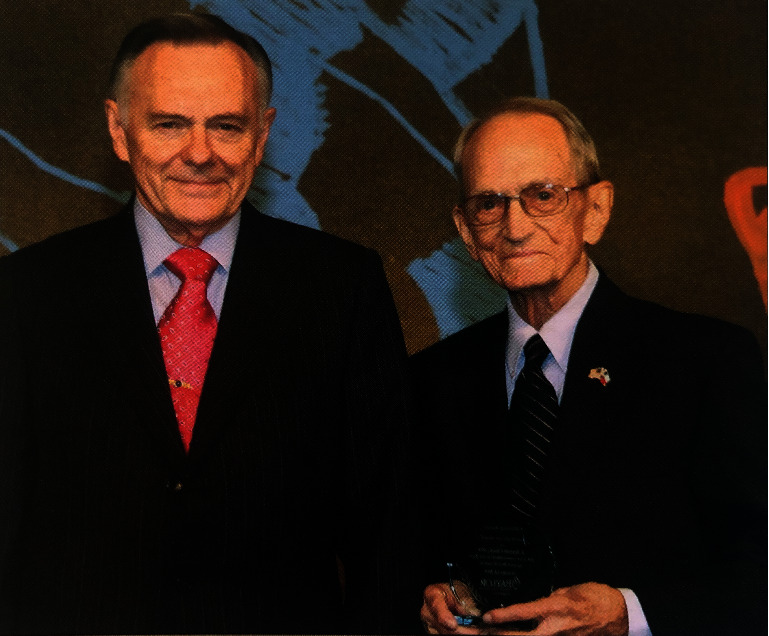
Dr. Ronald C. Jones, director of the Breast Surgical Oncology Fellowship at Baylor University Medical Center from 1999 to 2014, with Dr. J. Harold Cheek. Dr. Cheek was honored with The Circle of Care Award by Baylor Health Care System Foundation for his contributions to breast surgery and women's health.

The breast fellowship at Baylor University Medical Center received SSO accreditation on November 17, 2007. From 1995 until 2014, the fellowship at Baylor University Medical Center was under the leadership of Dr. Ronald Coy Jones, who also served as chairman of the Department of Surgery. Dr. Jones' role as the National Chairman of the Field Liaison Program helped hospitals become approved by the American College of Surgeons as accredited cancer hospitals, increasing the number of approved hospitals by 20%. In addition, his experiences as chairman of the executive committee of the Commission on Cancer, as well as vice chairman of the Commission on Cancer, further enabled Baylor's growth and promoted a strong breast surgical oncology fellowship, which now has trained over 30 fellows. As director of the fellowship, Dr. Jones honored the legacy of Dr. Cheek for his contributions to breast surgery, having him serve on multiple committees and maintain an active role in the department (Figure 4) [25].

Several studies have examined the effectiveness of breast surgical oncology fellowship regarding outcomes. An extensive review of the literature published in 2015 reported improved patient satisfaction and quality of life when treatment was performed by surgeons who specialized in breast surgery. Regarding breast cancer outcomes, the authors reported improved survival rates in patients who were treated by breast surgical oncology specialists [19]. A recent study published in 2020 reported an association between breast surgical specialization and improved long-term patient reported outcomes in cancer patients [26].

## 9. 2004–2011: Changes in Mastectomy Patterns: The Contralateral Prophylactic Mastectomy

Although there is no significant survival benefit to low-to-average risk patients who undergo contralateral prophylactic mastectomy (CPM), there has been an estimated increase in CPM rates in the United States since 2004. Interestingly, this has been associated with a decline in the proportion of breast conservation surgery. Data from the National Cancer Database illustrated that the annual rate of CPM increased by 14% with a decline in breast conservation surgery by 2% per year from 2005 to 2011 [27]. Studies have attempted to elicit the drivers of the trend toward CPM. Treatment factors including MRI at diagnosis, frequency of prior negative breast biopsies, and availability of immediate breast reconstruction are associated with increased CPM. Demographic factors, including younger age, white, or nonblack ethnic origin, and private health insurance are also commonly cited [28]. Evidence also suggests that the utilization of CPM is influenced by emotional factors, specifically the patient's desire to have peace of mind and reduce anxiety about experiencing a subsequent breast cancer diagnosis [29]. Regional factors also influence the use of CPM, with high rates in the southwest and low rates in the northeastern United States.

## 10. 2014: De-escalation of Surgical Care

A trend toward de-escalation of surgical care began in 2014 when the SSO and American Society for Radiation Oncology released new guidelines on the margins for breast-conserving surgery with whole breast irradiation in stages I and II invasive breast cancer [30]. The guidelines were based on the results of a meta-analysis, which included over 28,162 patients, which found that the vast majority or reexcisions were unnecessary, as disease control was excellent for women with early stage-disease when radiation and hormonal therapy and/or chemotherapy were added to the treatment plan. Thus, the number of reexcisions for early-stage breast cancer significantly decreased [30].

This trend continued when Dr. Armando Giuliano published data from the landmark American College of Surgeons Oncology Group Z0011 clinical trial, challenging the routine use of axillary lymph node dissection. The study showed that patients with tumors less than five centimeters and no palpable axillary adenopathy, who were found to have less than three sentinel nodes containing metastases, did not require axillary dissection, as there was no difference in overall survival compared with sentinel lymph node dissection alone [31]. Continued improvements in the understanding of adjuvant treatment, including chemotherapy, radiotherapy, and hormonal therapy, enable less invasive surgeries, minimizing the morbidity of axillary surgery.

## 11. 2015: The Campaign for Breast Reconstruction Awareness

Improvements in access and quality of breast reconstruction are closely linked to the higher utilization rates of CPM on a population level [32]. The United States had been experiencing a rise in both immediate and delayed breast reconstruction over the prior decade. The Implementation of the Women's Health and Cancer Rights Act in 1998 required payers to provide benefits for mastectomy-related services including all stages of reconstruction and symmetry procedures. In 2015, the Congress passed the Breast Cancer Patient Education Act to inform and educate breast cancer patients about the availability and coverage of breast reconstruction, prostheses, and other options [33]. Furthermore, the Plastic Surgery Foundation and the American Society of Plastic Surgeons have developed The Breast Reconstruction Awareness Campaign, with the goal to educate, engage, and empower women to make the decision that is best for them following a diagnosis with breast cancer [34]. These and similar efforts have contributed to the upsurge in the proportion of women obtaining breast reconstruction, with rates as low as 8% in 1995 to recently published rates above 50% in the American College of Surgeons National Surgical Quality Improvement Program data reported in 2014 [35]. The literature supports the psychologic, social, emotional, and functional benefits of breast reconstruction. The Michigan Breast Reconstruction Outcomes Study confirmed that that the general psychosocial benefits of breast reconstruction extend long term [36]. In addition to postmastectomy reconstruction, the usage of oncoplastic procedures in the setting of breast conversation is also increasing. Shaitelman et al. have examined the role of oncoplastic surgery in the management of breast cancer and how oncoplastic procedures may have an impact on oncologic treatments [37].

## 12. 2016–Present: The Movement Continues

Breast surgery has continued to grow and expand over the past several years. The National Accreditation Program for Breast Cancer (NAPBC) was developed as an outgrowth of the Cancer Programs of the American College of Surgeons and the Commission on Cancer. Accredited NAPBC programs adhere to standards of care, organize data collection, and work with the Commission on Cancer to improve patient survivorship and quality of life.

With earlier breast cancer detection and increased availability of effective treatments, most individuals diagnosed with breast cancer will experience long-term survival, leading to advancements in breast cancer survivorship. Survivorship care and services are aimed at managing issues that prevail following the completion of cancer treatment in the absence of active disease. A large area of focus is the adverse health consequences of breast cancer treatment, including cardiovascular effects, menopausal symptoms, sexual dysfunction, infertility, bone density loss, chronic pain, lymphedema, fatigue, cognitive changes, sleep problems, psychological symptoms, secondary malignancies, and financial toxicity including loss of employment [38].

Research and clinical trials, including those investigating topics such as nipple sparing mastectomy and the impact of the coronavirus disease 2019 pandemic on breast cancer patient treatment levels, are currently active across multi-institutions. More research is being pursued on the changes in body image associated with breast cancer and ways of protecting patient body image throughout diagnosis and treatment [39]. Various public figures and campaigns, along with the increasing number of support groups, strive to provide women with a sense of community and comfort, emphasizing that they are not alone in their diagnosis or breast cancer journey. Surgeons such as Dr. Harold P. Freeman have published studies on the racial disparities in breast cancer survival rates and the need to focus on early diagnosis [40]. Stemming from this, there are now various initiatives such as “Touch, The Black Breast Cancer Alliance” and “The Campeonas Project” that have been developed to raise breast cancer awareness in diverse groups and drive the collaborative efforts of survivors, advocates, advocacy organizations, healthcare professionals, researchers, and pharmaceutical companies [41].

Given the previously mentioned topics, the future of the breast surgical oncology fellowship has opportunities for growth and expansion. As the ASBrS has added the areas of Breast Ultrasound and Oncoplastic Surgery Certification to its accreditation programs, many fellowships are taking time to educate trainees in these fields. Other current areas of interest include sensation preserving mastectomies, circulating free DNA as a tumor marker, new imaging modalities for ER-positive cancers, the aforementioned survivorship factors, and the push for “shared decision making” between patient and surgeon.

## 13. International Influence and Implications

Although the present review focuses on American breast surgery and the breast surgical oncology fellowship, it should be noted that breast cancer is an international disease with international influences and training programs. While the trials of Dr. Fisher supported breast-conserving therapy in the USA, studies performed in Milan led by Dr. Veronesi from 1981 to 2002 supported the same. This led to a decline in the proportion of women receiving a mastectomy for early staged disease to only 18.6% in the European countries of Italy, Belgium, Germany, and Switzerland [42]. In 2005, data was published from the first clinical trial on the sentinel lymph node biopsy conducted at the European Institute of Oncology in Milan, supporting sentinel lymph node biopsy with no further axillary treatment if the node was negative. After the publication of the Z0011 trial, the International Breast Cancer Study Group Trial (IBCSG) 23-01 supported that the axillary dissection should be avoided in patients with minimal sentinel node involvement [43].

As far as specialization, the United Kingdom classifies breast surgery as a special interest within general surgery. Certification in breast surgery as a distinct entity is not yet established; however, the Fellowship of the Royal College of Surgeons (FRCS) examination allows the candidate to specify a breast special interest wherein the exam focuses on breast surgery to a higher level [44]. Progress towards specialization in the UK has seen most UK breast surgeons move away from emergency surgery and the establishment of a National Oncoplastic Training fellowship in 2002. Nearly 200 fellows have now completed the program, and most UK breast units now provide oncoplastic services, so women may undergo both oncologic and reconstructive surgery in a seamless way [44]. In Australia and New Zealand, a one- or two-year fellowship in breast surgery has been developed. The first year of the program covers the skills such as mastectomy and surgery of the axilla, while the second year covers oncoplastic and reconstructive procedures. [44].

## 14. Conclusion

Over the past 50 years, breast cancer surgery has evolved from dark shadows of the Halsted radical mastectomy to a public awareness campaign of surgical options, aesthetically pleasing reconstruction, and the development of the breast surgery specialty and fellowships. As further scientific and technologic advancements are made, the opportunities for breast surgeons will continue to expand. The scientific, political, and social influences mentioned in this article are just several of the historic factors that have paved the way for the modern-day breast surgeon. It is now our responsibility to continue the breast surgery movement, ultimately striving to improve the lives of breast cancer patients worldwide and to make the next 50 years as pivotal as the past.

## Data Availability

No data were used to support this study.
